# Naturally Acquired Binding-Inhibitory Antibodies to *Plasmodium vivax* Duffy Binding Protein in Pregnant Women Are Associated with Higher Birth Weight in a Multicenter Study

**DOI:** 10.3389/fimmu.2017.00163

**Published:** 2017-02-17

**Authors:** Pilar Requena, Myriam Arévalo-Herrera, Michela Menegon, Flor E. Martínez-Espinosa, Norma Padilla, Camila Bôtto-Menezes, Adriana Malheiro, Dhiraj Hans, Maria Eugenia Castellanos, Leanne Robinson, Paula Samol, Swati Kochar, Sanjay K. Kochar, Dhanpat K. Kochar, Meghna Desai, Sergi Sanz, Llorenç Quintó, Alfredo Mayor, Stephen Rogerson, Ivo Mueller, Carlo Severini, Hernando A. del Portillo, Azucena Bardají, Chetan C. Chitnis, Clara Menéndez, Carlota Dobaño

**Affiliations:** ^1^ISGlobal, Barcelona Ctr. Int. Health Res. (CRESIB), Hospital Clínic – Universitat de Barcelona, Barcelona, Catalonia, Spain; ^2^Caucaseco Scientific Research Center, Universidad del Valle, Cali, Colombia; ^3^Istituto Superiore di Sanità, Rome, Italy; ^4^Fundação de Medicina Tropical Dr. Heitor Vieira Dourado, Manaus, Amazonas, Brazil; ^5^Instituto Leônidas e Maria Deane (ILMD/Fiocruz Amazonia), Amazonia, Brazil; ^6^Centro de Estudios en Salud, Universidad del Valle de Guatemala, Guatemala City, Guatemala; ^7^Universidade do Estado do Amazonas, Manaus, Amazonas, Brazil; ^8^Instituto de Ciências Biológicas, Universidade Federal do Amazonas, Manaus, Brazil; ^9^International Center for Genetic Engineering and Biotechnology, Delhi, India; ^10^Papua New Guinea Institute of Medical Research, Madang, Papua New Guinea; ^11^Macfarlane Burnet Institute of Medical Research, Melbourne, VIC, Australia; ^12^Walter and Eliza Hall Institute, Parkville, VIC, Australia; ^13^Medical College Bikaner, Bikaner, Rajasthan, India; ^14^Centers for Disease Control and Prevention, Division of Parasitic Diseases and Malaria, Malaria Branch, Atlanta, GA, USA; ^15^University of Melbourne, Melbourne, VIC, Australia; ^16^ICREA, Barcelona, Spain

**Keywords:** malaria in pregnancy, vivax, falciparum, immunity, antibodies, T cell, cytokines, PvDBP

## Abstract

A vaccine to eliminate malaria would need a multi-stage and multi-species composition to achieve robust protection, but the lack of knowledge about antigen targets and mechanisms of protection precludes the development of fully efficacious malaria vaccines, especially for *Plasmodium vivax* (Pv). Pregnant women constitute a risk population who would greatly benefit from a vaccine preventing the adverse events of *Plasmodium* infection during gestation. We hypothesized that functional immune responses against putative targets of naturally acquired immunity to malaria and vaccine candidates will be associated with protection against malaria infection and/or poor outcomes during pregnancy. We measured (i) IgG responses to a large panel of Pv and *Plasmodium falciparum* (Pf) antigens, (ii) the capacity of anti-Pv ligand Duffy binding protein (PvDBP) antibodies to inhibit binding to Duffy antigen, and (iii) cellular immune responses to two Pv antigens, in a subset of 1,056 pregnant women from Brazil, Colombia, Guatemala, India, and Papua New Guinea (PNG). There were significant intraspecies and interspecies correlations for most antibody responses (e.g., PfMSP1_19_ versus PfAMA1, Spearman’s rho = 0.81). Women from PNG and Colombia had the highest levels of IgG overall. Submicroscopic infections seemed sufficient to boost antibody responses in Guatemala but not antigen-specific cellular responses in PNG. Brazil had the highest percentage of Duffy binding inhibition (*p*-values versus Colombia: 0.040; Guatemala: 0.047; India: 0.003, and PNG: 0.153) despite having low anti-PvDBP IgG levels. Almost all antibodies had a positive association with present infection, and coinfection with the other species increased this association. Anti-PvDBP, anti-PfMSP1, and anti-PfAMA1 IgG levels at recruitment were positively associated with infection at delivery (*p*-values: 0.010, 0.003, and 0.023, respectively), suggesting that they are markers of malaria exposure. Peripheral blood mononuclear cells from Pv-infected women presented fewer CD8^+^IFN-γ^+^ T cells and secreted more G-CSF and IL-4 independently of the stimulus used *in vitro*. Functional anti-PvDBP levels at recruitment had a positive association with birth weight (difference per doubling antibody levels: 45 g, *p*-value: 0.046). Thus, naturally acquired binding-inhibitory antibodies to PvDBP might confer protection against poor outcomes of Pv malaria in pregnancy.

## Introduction

According to the World Health Organization, 438,000 people died from malaria in 2015 mostly attributed to *Plasmodium falciparum* (*Pf*) infection in children (1) although increased numbers of severe malaria cases caused by *Plasmodium vivax* (*Pv*) have been reported in recent years (2–6). Naturally acquired immunity to clinical malaria develops with age and exposure to infections, and it is classically considered to rely on antibodies (7) though cellular memory responses to malaria antigens have also been implicated in immunity (8). Thus, immune responses against all *Pv* and/or *Pf* human stages have been reported, i.e., sporozoites (9, 10), merozoites (11, 12), asexual intraerythrocytic stages (13, 14), and gametocytes (15). Of note, sterile immunity is never acquired even in areas of high transmission, with adults having asymptomatic infections with low parasitemias (16) often only detected by PCR (17).

Despite this natural acquisition of immunity, adult pregnant women are more susceptible to the negative consequences of malaria infection than non-pregnant adults, and both *Pv* and *Pf* infections have been associated with poor pregnancy outcomes (18, 19). Nevertheless, immune mediators associated with susceptibility and clinical outcomes of malaria during pregnancy are not fully understood, especially for *Pv* infection. In the case of *Pf*, a parasite variant expressing the VAR2CSA protein on the surface of infected erythrocytes, to which primigravidae pregnant women are not immune as they have not been exposed before pregnancy, may accumulate in the placenta (20). Indeed, antibodies to VAR2CSA have been related to protection against or exposure to *Pf* malaria in pregnancy (20, 21), as well as protection against poor pregnancy outcomes (22, 23).

A *Pv* ligand for the placenta (similar to VAR2CSA) has not been identified thus far, but we recently reported a positive association between antibody levels against two *Pv* VIR proteins with 19 and 26% protein homology to VAR2CSA and birth weight (BW), respectively (24). Actually, there is controversy about whether *Pv* has cytoadhesive properties at all, although we have found placental *Pv* monoinfections in Papua New Guinea (PNG) (25). *Pv* infects human red blood cells mainly through interaction between the *Pv* ligand Duffy binding protein (PvDBP) and its receptor on reticulocytes, the Duffy antigen receptor for chemokines (DARC) (26). PvDBP, specifically the binding domain referred to as region II (PvDBPII), is a major vaccine candidate (27). Naturally acquired and experimentally induced antibodies to PvDBPII inhibit parasite invasion *in vitro* (28) and protect against *Pv* infection in children in a high transmission area of PNG (29) and clinical *Pv* malaria in adults in a low-transmission area in Brazil (BR) (30), supporting PvDBPII as a leading vaccine candidate. Additional characterization of naturally acquired immune responses to PvDBP and other *Pv* antigens during pregnancy is needed to identify those responses that may mediate protection in this condition and guide antigen selection for vaccine development.

Experts agree that a vaccine to eliminate malaria would need to include antigens from both *Pf* and *Pv* parasites (31). Furthermore, a multi-stage and multi-strain vaccine inducing both antibody and cellular immune responses would likely be required to achieve robust protection against malaria in areas of different endemicity. Here, we present a comprehensive longitudinal study of naturally acquired antibody responses to nine *Pv* antigens, including the only two vaccine candidates in clinical development: circumsporozoite protein (PvCSP) and PvDBP (32). In addition, functional capacity of anti-PvDPB and T cell responses to PvDBP and one merozoite surface protein (PvMSP1_19_) were assessed, as well as antibody responses to six *Pf* antigens. Women from five malaria endemic countries in Latin America, Asia, and the Pacific where *Pv* and *Pf* coexist were enrolled for this immune profiling, enabling us to compare responses among areas with different malaria transmission characteristics where diverse *Plasmodium* strains circulate. To our knowledge, this is the first study of this scope and magnitude conducted in a multi-country cohort of women during and after pregnancy.

## Materials and Methods

### Study Design and Population

This study was part of the PregVax project (FP7-HEALTH-201588, www.pregvax.net), which studied the burden, impact, immune responses, and pathophysiology of *Pv* in pregnancy between 2008 and 2012 in five endemic countries: BR, Colombia (CO), Guatemala (GT), India (IN), and PNG. Approximately 2,000 women per country were enrolled at the first visit at the antenatal clinic (recruitment) and followed up until delivery. In all visits, hemoglobin (Hb) levels, *Pv* and *Pf* parasitemias by blood smear and malaria symptoms were assessed. Giemsa-stained thick and thin blood slides were read onsite following WHO standard quality-controlled procedures, and external validation of a subsample of blood slides was done at the Hospital Clinic and at the Hospital Sant Joan de Deu, in Barcelona, Spain. BW was recorded. Women with a positive smear were treated according to national guidelines, except in PNG where blood smears could not be read at the moment of the visit for logistical reasons (only symptomatic women were thus treated after confirmation of infection by rapid diagnostic test).

From the PregVax cohort, 10% of women were randomly allocated to the “immunology cohort” and were followed up with one visit at least 10 weeks after delivery (postpartum group). At recruitment, delivery and postpartum visits, a venous blood sample (5–10 mL) was collected aseptically in heparinized tubes. Submicroscopic *Pv* and *Pf* infections were also determined in a random subsample by real-time PCR, except for the Indian samples, where only *Pv* infection was examined. Additionally, blood samples (10 mL) were collected from 39 malaria naïve donors at the blood bank in Hospital Clinic (Barcelona, Spain) and used as negative controls.

### Ethics Statement

The protocol was approved by the Hospital Clinic Ethics Review Committee (CEIC, Barcelona, Spain), the CDC IRB (USA), and the national and/or local ethics committees of each site: the Universidad del Valle de Guatemala Ethics Review Committee (CE-UVG, GT); the *Comité Institucional de Revisión de Ética Humana, Facultad de Salud, Universidad del Valle de Cauca* (Cali, CO); the *Comissâo Nacional de Ética em Pesquisa* (CONEP, BR); the *Comitê de Ética em Pesquisa da Fundação de Medicina Tropical do Amazonas* (FMT-AM, Manaus, BR); the Ethics Committee, Sardar Patel Medical College and A.G. Hospitals (Bikaner, Rajasthan, IN); and the Medical Research Advisory Committee in PNG (MRAC 08.02). Written informed consent was obtained from all study participants.

### Processing of Plasma and Peripheral Blood Mononuclear Cells (PBMCs)

Plasma was separated by centrifugation and stored at −80°C. Blood cells from PNG and Spain were further fractioned in a density gradient medium (Histopaque^®^-1077, Sigma-Aldrich) to obtain PBMCs and stored in liquid nitrogen. Samples from GT, CO, BR, and PNG were analyzed at ISGlobal (Barcelona, Spain) while plasmas from IN were analyzed in Delhi.

### Recombinant Proteins and Synthetic Peptides

Unless otherwise specified, recombinant proteins were cloned with an *Escherichia coli* system using genomic DNA from different *Plasmodium* strains as template. Pv200L (amino acid residues 121–416 of PvMSP1) was amplified from genomic DNA obtained from a Colombian *Pv*-infected patient (33). PvDBP (receptor-binding domains—RII) was cloned using *Pv* Sal-I as template (34). PvMSP1_19_ (19 kDa C-terminal region, residues 1639–1729) was expressed using the Belem strain as template (35) and PfMSP1_19_ with the 3D7 strain (36). PfAMA1 (N-terminal ectodomain) was cloned using genomic DNA from the *Pf* 3D7 strain (37). PfEBA_175_ (the receptor-binding domain—PfF2) was cloned from the *Pf* CAMP strain (38). The *Pf* A4 strain was used as template for the VAR2CSA DBL3X domain (39) and 3D7 strain for the DBL5ε (40) and DBL6ε (41) VAR2CSA domains.

PvMSP1-N (fragment 170-675), full-length PvCSP, and full-length PvMSP5 were expressed with a glutathione S-transferase (GST) tag in a cell-free wheat germ system as reported previously (11). Expressed proteins were purified on GST SpinTrap purification columns (GE Healthcare), and eluted proteins were dialyzed in phosphate buffered saline (Tube-O-DIALYZER™, GBiosciences). GST was also expressed separately for immune-reactivity control. The production of the PvCSP-N (residues 20–96), PvCSP-C (residues 301–372), and PvCSP-R (three tandem-repetitions of the residues 96–104) peptides has been described elsewhere (9). Five PvDBP peptides [VNNTDTNFH(R/S)DITFR, LYLKRKLIYDAAVEG, and LIYDAAVEGDLL(L/F)KL] containing immunogenic epitopes previously published (42) were synthesized at >80% purity by Peptide 2.0 (Chantilly, VA, USA) for cellular stimulation assays.

### Quantification of IgG Antibodies

Measurement of plasma IgG antibodies was performed by multiplex suspension array using the Luminex™ technology, as described before (24). Briefly, 1.1–1.4 million MagPlex^®^ magnetic-carboxylated microspheres (Luminex Corporation, TX, USA) with different spectral signatures were covalently coated with 3 µg of each protein/peptide, following the manufacturer’s instructions. Protein-coupled beads were quantified in a Guava^®^ Flow Cytometer (Millipore) and mixed in equal amounts. A unique batch of microspheres was prepared for the whole study, including the samples analyzed in IN. Approximately 1,000 beads per analyte were incubated with each plasma (1:100 dilution) in duplicates, and subsequently with antihuman IgG-biotin (Sigma-Aldrich), followed by streptavidin-conjugated R-PE (Fluka, Madrid, Spain). Beads were acquired on the BioPlex100 system (Bio-Rad, Hercules, CA, USA), and results were expressed as median fluorescence intensity (MFI) of duplicates. Value against GST alone was subtracted from correspondent proteins. Raw GST data have been previously published (24). Cross-reactivity was ruled out in a pilot study analyzing a subset of plasmas in singleplex and multiplex (not shown). Samples in IN were analyzed with identical protocols and instruments.

### Detection of PvDBP-Binding Inhibitory Antibodies

A functional assay was carried out to study the capacity of anti-PvDBP antibodies to inhibit the binding of this protein to its receptor DARC, as previously described (43). Only plasma samples from responder women (anti-PvDBP MFI values above the mean + 3 SD of Spanish naïve controls) at recruitment were included in the functional assays. Ten microliters of plasma from each donor were preincubated with 0.01 µg/mL of PvDBP for 1 h at RT before adding to Bioplex plates containing 1,000 Luminex beads pre-coupled to DARC-Fc (N-terminal extracellular 60 amino acids of DARC fused to Fc region of human IgG). Bound PvDBP was detected with anti-PvDBP, followed by anti-human IgG-biotin and streptavidin-conjugated R-PE. A standard curve using increasing amount of PvDBP was prepared to establish concentration of PvDBP bound to DARC-Fc. Percent inhibition was calculated as % Inhibition = (1 − observed PvDBP conc./expected PvDBP conc.) × 100.

### Cellular Stimulation Assays

Except where indicated, all reagents were purchased from BD Biosciences, and all antibodies were monoclonal. PBMCs were thawed and rested for 10–12 h. Only samples with viability >70% (Guava ViaCount Reagent, Millipore) were used for assays. Half a million cells per well were resuspended in *RPMI-1640* medium plus 10% fetal bovine serum (culture medium) and incubated in the presence of a pool containing the five PvDBP peptides (5 µg/mL) or PvMSP1_19_ (10 µg/mL). Culture medium was used as negative control. Anti-CD3 stimulation was the positive control (24). After 12 h, an aliquot of 30 µL of culture medium supernatant was collected to measure secreted cytokines and replaced with media containing GolgiPlug™ for an additional (4 h) incubation. PBMCs were stained with LIVE/DEAD^®^ Fixable Violet Dead (Life Technologies), anti-CD14 Pacific Blue (clone M5E2), anti-CD19 Horizon™ V450 (clone HIB19), anti-CD4 allophycocyanin (clone RPA-T4), and anti-CD8 Peridinin Chlorophyll Protein Complex (PerCP, clone SK1). After washing, cells were fixed and permeabilized with Cytofix/Cytoperm™ and incubated with anti-CD3 phycoerythrin (PE)-Cy™7 (clone SK7), anti-interferon (IFN)-γ PE (clone 25723.11), and anti-CD69 fluorescein isothiocyanate (clone L78). Cells were acquired in an LSRFortessa flow cytometer, and data were analyzed by FlowJo (FlowJo LLC, OR, USA). The gating strategy was developed as previously (24). Supernatants were frozen at −80°C until Luminex analysis with the Cytokine Magnetic 30-Plex Panel (Invitrogen), according to the manufacturer’s instructions.

### *Plasmodium* spp. Detection by PCR

Samples from BR, CO, GT, and half samples from PNG were analyzed at the Istituto Superiore di Sanità (Rome, Italy), as described (44). Positivity for each species was established as a cycle threshold <45, according to negative controls. *Pv* diagnosis for IN samples was performed in Delhi following Rome’s protocol adapted for the instrument sensitivity (third step amplification 72°C for 25 s instead of 72°C for 5 s). Approximately half of PNG samples were analyzed for submicroscopic infections in Madang, following a similar protocol to Rome’s (45), except that positivity for each species was established as cycle threshold <40, according to negative controls. DNA was extracted from whole blood-spot samples on filter paper.

### Definitions and Statistical Methods

Any *Plasmodium* infection was defined as a positive smear and/or positive PCR. A positive antibody response (responder) was considered as MFI values above the mean + 3 SD of Spanish controls. To evaluate the differences in antibody levels among countries, a one-way ANOVA test was used followed by Bonferroni pairwise correction. To study the association between antibody levels and pregnancy variables, univariate (only adjusted for country of origin) and multivariate linear regression models were estimated with the following variables: country, age, gestational age, gravidity (number of previous gestations), and *Pv* or *Pf* infection during pregnancy (considering past or present infections but not future–delivery—infections). The correlation between IgG responses to different antigens was evaluated with the Spearman’s rank test. The association between IgG levels at recruitment and malaria infections at delivery was evaluated with logistic regression models. The association between antibody responses and pregnancy outcomes, i.e., Hb levels at delivery and BW, was analyzed using univariate and multivariate linear regression models, adjusted by country, Hb at recruitment, gestational age at recruitment, age, gravidity, and past or present *Plasmodium* infection during pregnancy. For the cellular and cytokine analyses, deviation from normality was tested using the skewness and kurtosis test. Because none of the variables except IL-13 presented a normal distribution, data were presented as medians, and comparisons between groups were done using the Mann–Whitney *U* test. The cytokine multiple comparisons were adjusted using the Bonferroni test. Cytokine/chemokine production in culture supernatants of unstimulated samples (medium) was not subtracted from the stimulated samples but shown side by side as it is possibly biologically relevant. Significance was defined at *p* < 0.05. Analyses and graphs were performed using Stata/SE 10.1 (College Station, TX, USA).

## Results

### Study Population

In total, 1,491 peripheral blood samples (794 at recruitment—first antenatal visit, 529 at delivery, and 168 postpartum) corresponding to 1,056 women were analyzed for IgG antibody responses against both *Pv* (six recombinant proteins and three peptides) and *Pf* (six recombinant proteins). Unfortunately many of our samples were not matched due to late attendance to antenatal clinics or low follow-up rate. The baseline study population characteristics and infection rates by country have been published previously (24).

For cellular assays, 46 samples (any gestational age including delivery, 18 *Pv* PCR negative, 28 *Pv* PCR positive) from the PNG pregnant cohort were included in the analyses. When cell counts were limited, stimulation with PvMSP1_19_ was the last priority, resulting in a lower sample size (12 *Pv* PCR negative and 15 *Pv* PCR positive).

### Antibody Responses to *Pv* Preerythrocytic Antigens

**Figure 1:**
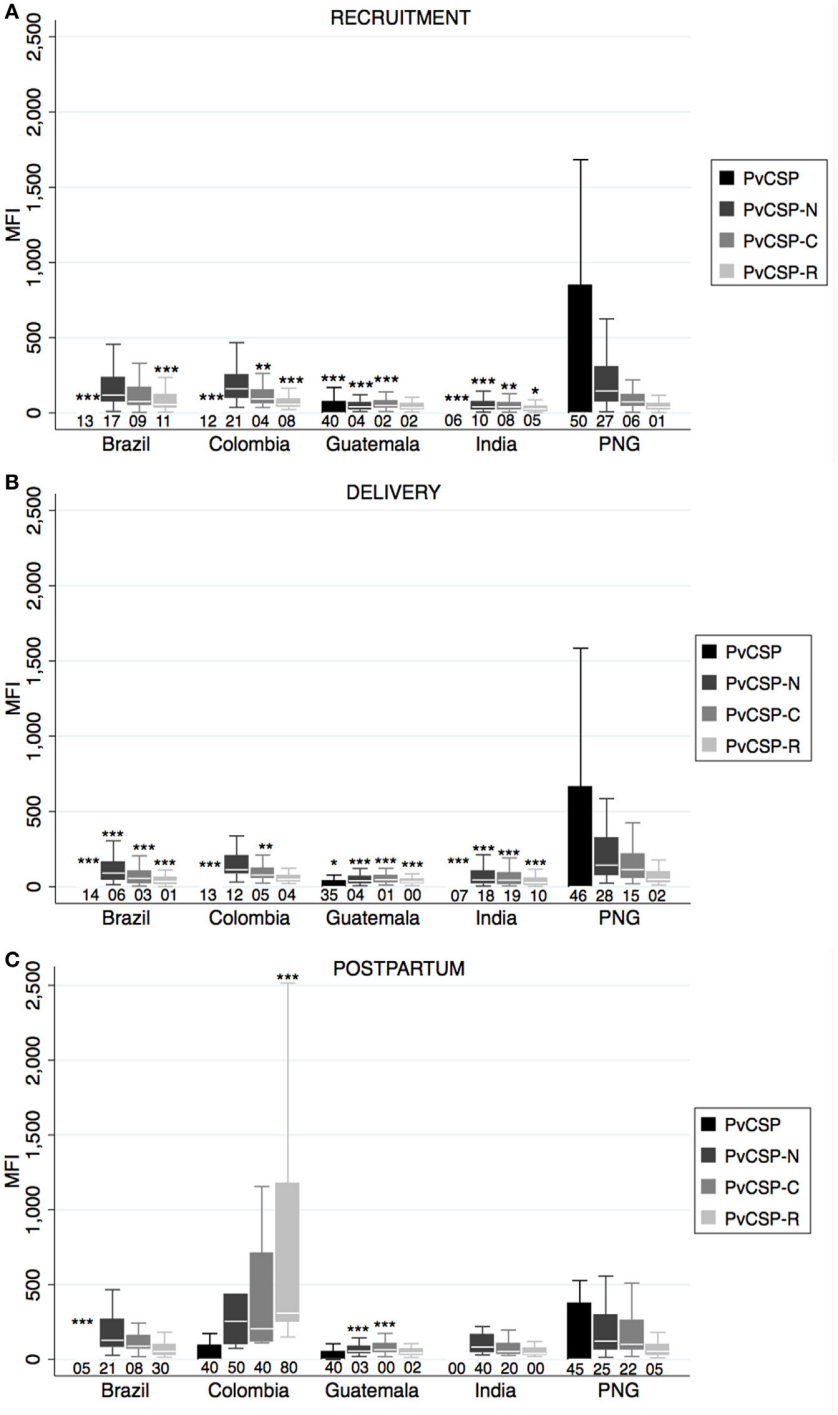
**Antibody responses against *Plasmodium vivax* preerythrocytic stage antigens**. Human IgG antibodies against *P. vivax* preerythrocytic stage antigens were detected by Luminex in peripheral plasma at recruitment **(A)**, delivery **(B)**, and postpartum **(C)**. Antibody responses are represented as median fluorescence intensity (MFI). Reactivity (MFI) against glutathione S-transferase was subtracted from MFI values of PvCSP. Median (white line), and 25th and 75th percentiles (lower and upper hinge, respectively) are represented in the box. Outside values are not displayed in the graphs. Numbers below boxes represent percentage of positive responses, calculated as number of plasmas with MFI value above mean + 3 SD of negative controls from Spain. Cutoff for India samples was calculated with negative samples analyzed at this site and therefore differed from cutoff used for the other sites. *p*-Values correspond to one-way ANOVA plus Bonferroni pairwise correction. *p < 0.05, **p < 0.01, ***p < 0.001. Only displayed differences versus Papua New Guinea (PNG). Sample size: recruitment, Brazil: 133, Colombia: 217, Guatemala: 173, India: 134, PNG: 137; delivery, Brazil: 75, Colombia: 117, Guatemala: 105, India: 98, PNG: 134; postpartum, Brazil: 38, Colombia: 10, Guatemala: 60, India: 5, PNG: 55.

**Figure 2:**
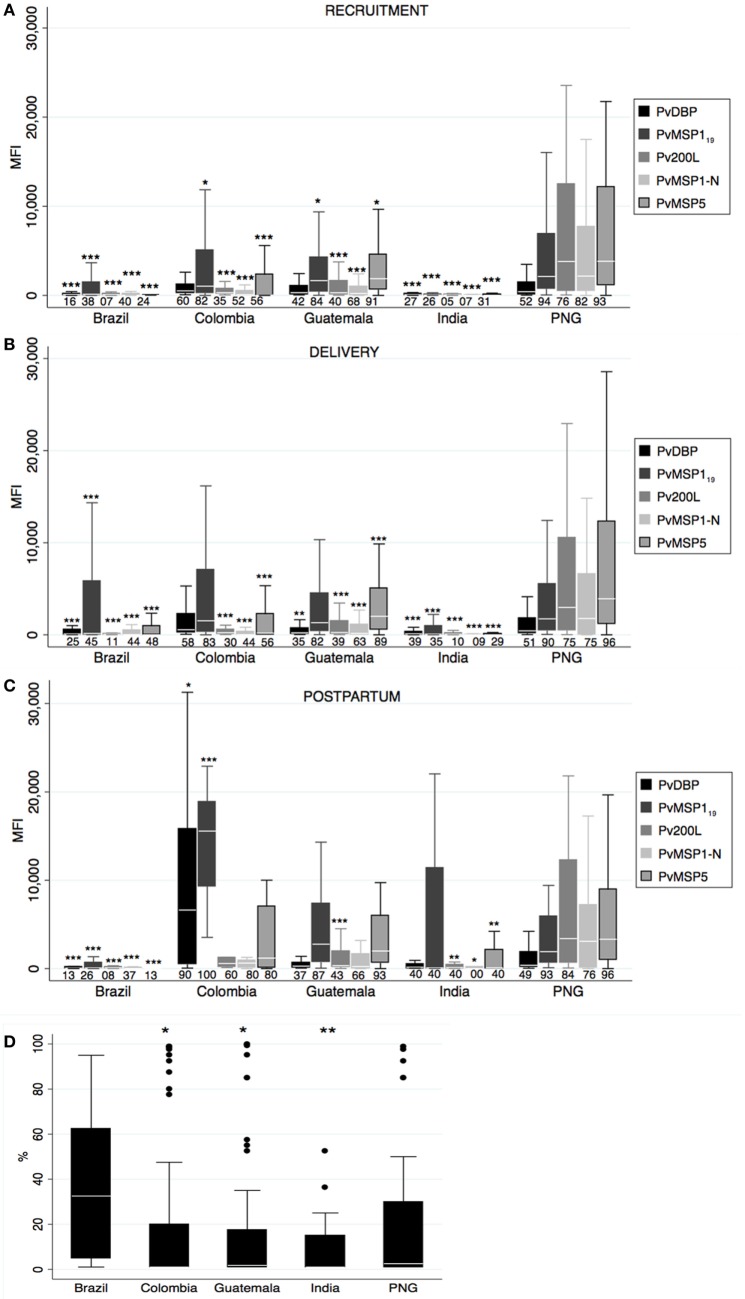
**Antibody responses against *Plasmodium vivax* merozoite stage antigens**. Human IgG antibodies against *P. vivax* merozoite stage antigens were detected by Luminex in peripheral plasma at recruitment **(A)**, delivery **(B)**, and postpartum **(C)**. Antibody response representation and sample size are as in Figure 1 (see Figure 1 legend). **(D)** Percentage of *Pv* ligand Duffy binding protein (PvDBP)-binding inhibition at recruitment. Sample size: Brazil: 18, Colombia: 71, Guatemala: 52, India: 34, and Papua New Guinea (PNG): 47. *p*-Values correspond to one-way ANOVA plus Bonferroni pairwise correction. *p < 0.05, **p < 0.01, ***p < 0.001

IgG MFI values against the four *Pv* preerythrocytic antigens differed among countries at all timepoints (one-way ANOVA *p* < 0.05). Overall, PNG had the highest levels of all anti-PvCSP antibodies at recruitment and delivery followed by CO; at postpartum CO had similar or higher levels (Figure 1). PvCSP protein was more reactive (seropositivity and/or MFI levels) than the three PvCSP peptides in PNG and GT, but they were recognized at similar levels in BR, CO, and IN. PvCSP-N was the most reactive of the three peptides in all countries and at all timepoints, except CO and BR at postpartum, where PvCSP-R was more seroprevalent (Figure 1). We also observed higher anti-PvCSP peptide levels at postpartum compared to recruitment and delivery, but this was not found for anti-PvCSP protein levels (Table S1 in Supplementary Material). In general and as expected, MFI and seroprevalence values for anti-PvCSP antibodies were low compared to merozoite antigens (Figure 2).

### Antibody Responses to *Pv* Merozoite Stage Antigens

Similar to anti-PvCSP responses, prevalence of anti-*Pv* merozoite antibodies differed among countries at all timepoints (one-way ANOVA *p* < 0.05), and PNG presented the highest magnitudes and prevalence at recruitment and delivery followed by CO (Figures 2A,B). In contrast to anti-PvCSP responses, women from GT presented remarkable anti-merozoite levels at all timepoints in spite of lower prevalence of patent malaria infections. Reactivity of each antigen varied across countries and timepoints; for instance antibody values for Pv200L and PvMSP1-N were lower than for PvDBP in BR, CO, and IN, but similar and higher in GT and PNG, respectively. The levels of PvDBP and PvMSP1_19_ antibodies in CO at postpartum were very high compared to the rest of countries (Figure 2C). We did not find differences in antibody levels between timepoints.

We also measured binding-inhibition percentage to Duffy antigen in the plasma of positive PvDBP responders at recruitment. Although BR had the lowest number of responders, the anti-PvDBP antibodies on those responders had the highest amount of inhibitory activity (one-way ANOVA *p* = 0.01, Figure 2D), with 8/18 individuals having more than 50% inhibitory activity.

### Antibody Responses to *Pf* Blood Stage Antigens

**Figure 3:**
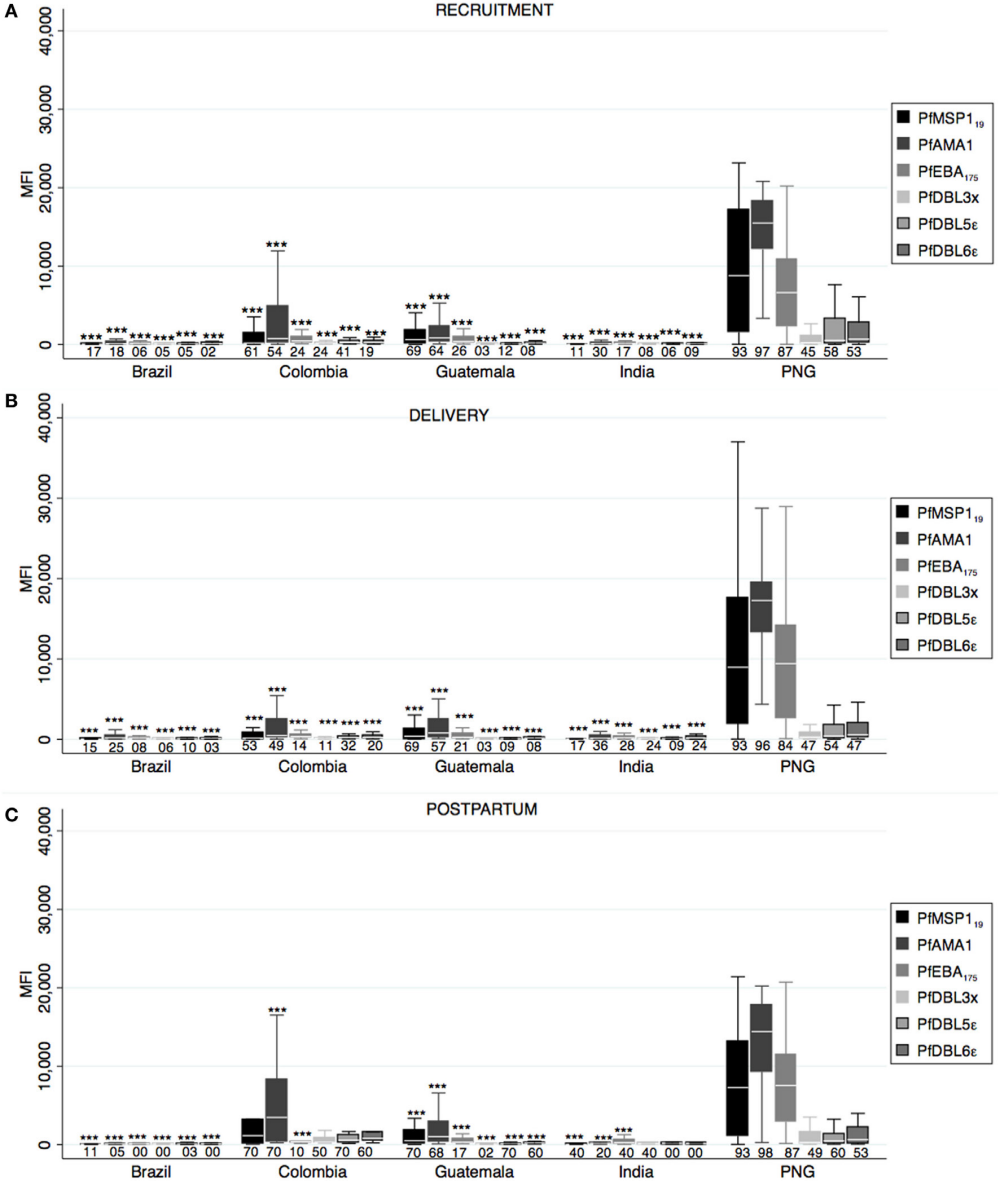
**Antibody responses against *Plasmodium falciparum merozoite and* erythrocytic stage antigens**. Human IgG antibodies against *P. falciparum* blood stage antigens were detected by Luminex in peripheral plasma at recruitment **(A)**, delivery **(B)**, and postpartum **(C)**. Antibody response representation and sample size are as in Figure 1 (see Figure 1 legend). *p*-Values correspond to one-way ANOVA plus Bonferroni pairwise correction. ***p < 0.001

Differential *Pf* antibody responses were observed between countries at all timepoints (one-way ANOVA *p* < 0.05), and PNG presented the highest magnitudes except for PfMSP1_19_, PfDBL3x, PfDBL5ε, and PfDBL6ε at postpartum that were similar in CO (Figure 3). CO presented the second highest anti-*Pf* antibody levels, while antibodies were almost absent in BR and IN. Of note, GT presented remarkable anti-*Pf* responses, similar in some cases to CO, despite low prevalence of *Pf* infection in our cohort. PfAMA1 antibodies had the highest MFI values (not so for seroprevalence) in all cases, and anti-PfAMA1 IgG levels in PNG were higher than any *Pv* antigen-specific antibodies (Figure 3). When we compared magnitude of antibody responses between timepoints, we found lower anti-PfMSP1_19_ at delivery and higher anti-PfDBL3x levels at postpartum compared to recruitment (Table S1 in Supplementary Material).

### Correlation of Antibody Levels

**Figure 4:**
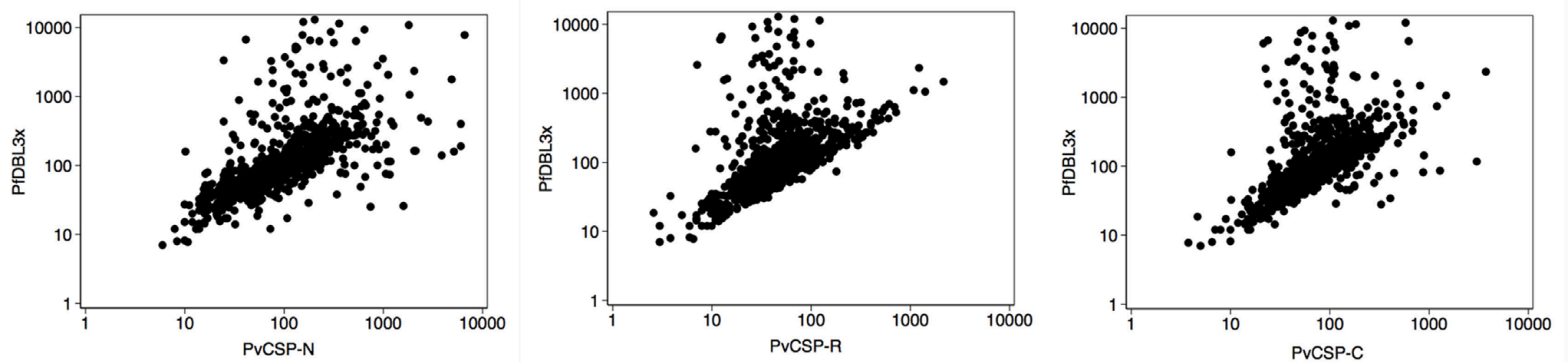
**Correlation of antibody responses**. Scatter plots show the distribution of values of mean fluorescence intensity in logarithmic scale for anti-PfDBL3x and anti-PvCSP-N, -R, and -C antibody levels. Samples corresponded to pregnant women of all countries at recruitment (*n* = 794).

To study the possibility of cross-reactivity, we analyzed the correlation between antibody levels (Table 1). There was a good correlation between the levels of antibodies of the same group (preerythrocytic, merozoite, and PfEMP1 family) except for PvCSP protein and peptides. The highest intraspecies correlation was observed between anti-PfMSP1_19_ and anti-PfAMA1 levels (Table 1). With regards to interspecies correlation, this was relatively high between *Pv* and *Pf* anti-merozoite antibody levels (Spearman’s rho ≥ 0.45 depending on the antibody, *p* < 0.0001 for them all, Table 1). Interestingly, we found a high correlation between levels of antibodies against the three PvCSP peptides and VAR2CSA domains, especially PfDBL3x (Spearman’s rho ≥ 0.64, *p* < 0.0001, Table 1; Figure 4). The percentage of PvDBP functional antibodies correlated poorly with the levels of all antibodies, except for anti-PvDBP, anti-PvMSP1-N, and anti-PvMSP5 (all *p* < 0.0001).

**Table 1:** **Correlation of antibody levels**.

	PvCSP-N	PvCSP-C	PvCSP-R	PvCSP	PvDBP	PvMSP1_19_	Pv200L	PvMSP1-N	PvMSP5	PvDBP-Inh	PfMSP1_19_	PfAMA1	PfEBA_175_	PfDBL3x	PfDBL5ε	PfDBL6ε		
PvCSP-N	1.00																	
PvCSP-C	0.71	1.00																
PvCSP-R	0.70	0.70	1.00															
PvCSP	0.12	0.12	0.06	1.00														
PvDBP	0.45	0.44	0.41	0.15	1.00													
PvMSP1_19_	0.31	0.34	0.26	0.24	0.67	1.00											0.0–|0.2|	
Pv200L	0.44	0.39	0.31	0.34	0.57	0.57	1.00										0.21–0.4	
PvMSP1-N	0.17	0.17	0.06	0.06	0.35	0.47	0.52	1.00									0.41–0.6	
PvMSP5	0.06	0.06	−0.01	−0.01	0.39	0.51	0.51	0.59	1.00								0.61–0.8	
PvDBP-Inh	0.08	0.08	0.03	0.03	0.22	0.20	0.07	0.22	0.25	1.00							0.81–1	
PfMSP1_19_	0.41	0.34	0.27	0.33	0.54	0.59	0.67	0.50	0.53	−0.06	1.00							
PfAMA1	0.33	0.26	0.17	0.31	0.51	0.58	0.66	0.50	0.56	0.07	0.81	1.00						
PfEBA_175_	0.35	0.26	0.19	0.29	0.51	0.45	0.64	0.42	0.47	−0.08	0.72	0.72	1.00					
PfDBL3x	0.76	0.68	0.64	0.17	0.55	0.40	0.54	0.24	0.18	0.07	0.54	0.50	0.55	1.00				
PfDBL5ε	0.63	0.56	0.49	0.21	0.53	0.42	0.58	0.28	0.25	0.12	0.54	0.53	0.54	0.79	1.00			
PfDBL6ε	0.53	0.45	0.40	0.25	0.49	0.42	0.57	0.28	0.30	0.07	0.55	0.55	0.60	0.73	0.68	1.00		

*Spearman’s correlation coefficient (rho, range 0–|1|) is displayed in the cells. Samples included in this analysis belonged to recruitment. *N* = 794, except for *Pv* ligand Duffy binding protein (PvDBP)-Inh (% of Duffy binding inhibition) where *N* = 223*.

### Association of Antibody Levels with Pregnancy Variables and Infection

Next, we assessed how different pregnancy variables affected the levels of antibodies. As expected, country of origin was significantly associated with all antibodies, both at recruitment and at delivery (not shown). In the adjusted regression, PfMSP1_19_ IgG responses had a positive association with age at delivery (proportional differences per 1 year increase in age = 1.04, 95% CI = 1.00–1.08, *p* = 0.042). PvMSP1-N antibody levels had a positive association with gravidity [proportional difference in antibody levels per belonging to category: (0 previous gestations) = 1; (1–3 previous gestations) = 2.06, 95% CI = 1.24–3.42; (4+ previous gestations) = 2.01, 95% CI = 0.87–4.63; *p* = 0.020] and with gestational age (proportional differences per 1 week increase in gestational age = 1.05, 95% CI = 1.02–1.08, *p* = 0.002) at recruitment. Most antibodies presented a positive association with current *Plasmodium* infection (infection status at time of sample collection), which was more prominent at recruitment than at delivery (Tables 2 and 3, data at delivery not shown). Of note, women with *Pf* and *Pv* coinfection had significantly more anti-PvMSP1_19_, anti-Pv200L, and anti-PvDBP (borderline difference for PvDBP) antibodies than women with *Pv* monoinfections (Table 2). Similarly, coinfected women had more anti-*Pf* antibodies than women with *Pf* infection alone in most cases (Table 3). However, sample size for coinfections was small, and these results should be considered cautiously.

**Table 2:** **Association of antibody levels against *P. vivax* at recruitment and infection status at recruitment**.

		Recruitment		
		Adj eff	95% CI	*p*
PvCSP	Pv−/Pf−	1.00		**0.039**
	Pv+/Pf−	2.26	1.18; 4.33	
	Pv−/Pf+	0.65	0.35; 1.22	
	Pv+/Pf+	1.62	0.27; 9.65	

PvCSP-N	Pv−/Pf−	1.00		0.130
	Pv+/Pf−	1.32	1.02; 1.72	
	Pv−/Pf+	1.10	0.85; 1.41	
	Pv+/Pf+	1.46	0.71; 3.00	

PvCSP-C	Pv−/Pf−	1.00		0.051
	Pv+/Pf−	1.32	1.05; 1.66	
	Pv−/Pf+	0.97	0.78; 1.21	
	Pv+/Pf+	1.62	0.86; 3.04	

PvCSP-R	Pv−/Pf−	1.00		**0.030**
	Pv+/Pf−	1.43	1.12; 1.81	
	Pv−/Pf+	0.99	0.79; 1.24	
	Pv+/Pf+	1.27	0.66; 2.46	

*Pv* ligand Duffy binding protein	Pv−/Pf−	1.00		**<0.001**
	Pv+/Pf−	2.42	1.65; 3.57	
	Pv−/Pf+	1.31	0.91; 1.90	
	Pv+/Pf+	2.83	0.98; 8.21	

PvMSP1_19_	Pv−/Pf−	1.00		**<0.001**
	Pv+/Pf−	2.67	1.60; 4.45	
	Pv−/Pf+	1.25	0.77; 2.03	
	Pv+/Pf+	15.67	3.84; 63.89	

Pv200L	Pv−/Pf−	1.00		**<0.001**
	Pv+/Pf−	2.40	1.57; 3.66	
	Pv−/Pf+	1.35	0.90; 2.02	
	Pv+/Pf+	4.15	1.29; 13.35	

PvMSP1-N	Pv−/Pf−	1.00		0.072
	Pv+/Pf−	2.60	1.09; 6.20	
	Pv−/Pf+	1.04	0.46; 2.37	
	Pv+/Pf+	6.94	0.65; 74.24	

PvMSP5	Pv−/Pf−	1.00		0.091
	Pv+/Pf−	2.40	1.02; 5.65	
	Pv−/Pf+	0.59	0.26; 1.31	
	Pv+/Pf+	2.53	0.25; 26.11	

*Adjusted effect (Adj eff) represents the fold increase in antibody levels [median fluorescence intensity (MFI)] per belonging to each category, after adjusting for the following variables: country of origin, age, gravidity (number of previous gestations), and gestational age. Pv: *Plasmodium vivax*. Pf: *Plasmodium falciparum*. N: Pv−/Pf− *N* = 672; Pv+/Pf− *N* = 52; Pv−/Pf+ *N* = 33; Pv+/Pf+ *N* = 5. Highlighted in bold if p-value < 0.05*.

**Table 3:** **Association of antibody levels against *P. falciparum* at recruitment and infection status at recruitment**.

		Recruitment		
		Adj eff	95% CI	*p*
PfMSP1_19_	Pv−/Pf−	1.00		**<0.001**
	Pv+/Pf−	1.49	0.93; 2.38	
	Pv−/Pf+	2.34	1.50; 3.66	
	Pv+/Pf+	6.06	1.67; 22.03	

PfAMA1	Pv−/Pf−	1.00		**<0.001**
	Pv+/Pf−	1.55	1.02; 2.33	
	Pv−/Pf+	1.87	1.27; 2.77	
	Pv+/Pf+	5.98	1.93; 18.52	

PfEBA_175_	Pv−/Pf−	1.00		**0.003**
	Pv+/Pf−	1.18	0.85; 1.63	
	Pv−/Pf+	1.48	1.09; 2.02	
	Pv+/Pf+	3.45	1.41; 8.44	

PfDBL3x	Pv−/Pf−	1.00		**<0.001**
	Pv+/Pf−	1.34	1.01; 1.78	
	Pv−/Pf+	1.56	1.19; 2.04	
	Pv+/Pf+	2.54	1.16; 5.55	

PfDBL5ε	Pv−/Pf−	1.00		**<0.001**
	Pv+/Pf−	1.61	1.15; 2.27	
	Pv−/Pf+	1.61	1.17; 2.23	
	Pv+/Pf+	3.07	1.20; 7.86	

PfDBL6ε	Pv−/Pf−	1.00		**<0.001**
	Pv+/Pf−	1.35	1.02; 1.80	
	Pv−/Pf+	1.76	1.35; 2.31	
	Pv+/Pf+	1.48	0.68; 3.23	

*Adjusted effect (Adj eff) represents the fold increase in antibody levels [median fluorescence intensity (MFI)] when comparing each infection group with the uninfected (Pv−/Pf−) one, after adjusting for the following variables: country of origin, age, gravidity (number of previous gestations), and gestational age. Pv: *Plasmodium vivax*. Pf: *Plasmodium falciparum*. N: Pv−/Pf− *N* = 672; Pv+/Pf− *N* = 52; Pv−/Pf+ *N* = 33; Pv+/Pf+ *N* = 5. Highlighted in bold if p-value < 0.05*.

### Association between Antibody Levels, Future Malaria and Delivery Outcomes

We also analyzed the association between the levels of antibody levels at recruitment in uninfected women and infection rates at delivery (future infection). Anti-PvDBP levels were positively associated with future *Pv* infection, and anti-PfMSP1_19_ and anti-PfAMA1 levels were positively associated with future *Pf* infection (Table 4). In addition, antibody levels against PfMSP1_19_ and PfAMA1 were associated with future *Pv* infection (Table 4).

**Table 4:** **Association of antibody levels at recruitment and infection status at delivery (future infection)**.

	Future *Pv* infection						Future *Pf* infection					
	Crude (adjusted by site)			Adjusted			Crude (adjusted by site)			Adjusted		
	OR	95% CI	*p*	OR	95% CI	*p*	OR	95% CI	*p*	OR	95% CI	*p*
PvCSP	1.00	0.84; 1.20	0.998	0.97	0.79; 1.18	0.744	1.29	0.99; 1.69	0.058	1.30	0.99; 1.70	0.056
PvCSP-N	1.46	0.94; 2.27	0.092	1.57	0.98; 2.51	0.058	1.31	0.75; 2.30	0.337	1.34	0.76; 2.35	0.309
PvCSP-C	1.05	0.57; 1.95	0.877	1.04	0.53; 2.04	0.902	1.10	0.49; 2.44	0.822	1.13	0.48; 2.64	0.778
PvCSP-R	1.09	0.57; 2.06	0.801	1.09	0.55; 2.16	0.800	0.85	0.36; 1.99	0.703	0.85	0.35; 2.09	0.725
PvDBP	**1.59**	**1.13; 2.24**	**0.008**	**1.64**	**1.13; 2.38**	**0.010**	1.00	0.61; 1.64	0.996	1.02	0.63; 1.67	0.925
PvMSP1_19_	1.09	0.81; 1.47	0.577	1.11	0.81; 1.54	0.511	1.13	0.76; 1.67	0.555	1.13	0.76; 1.68	0.534
Pv200L	0.92	0.69; 1.24	0.602	0.93	0.68; 1.28	0.660	1.27	0.81; 2.00	0.296	1.30	0.82; 2.06	0.259
PvMSP1-N	1.13	0.94; 1.35	0.199	1.19	0.96; 1.46	0.110	1.06	0.84; 1.33	0.642	1.07	0.85; 1.35	0.558
PvMSP5	1.02	0.84; 1.23	0.837	1.04	0.84; 1.27	0.740	1.17	0.86; 1.58	0.314	1.16	0.84; 1.58	0.368
PvDBP-inhibition	1.24	0.84; 1.84	0.272	1.38	0.88; 2.17	0.162	0.99	0.48; 2.02	0.973	0.75	0.40; 1.39	0.359
PfMSP1_19_	**1.41**	**1.05; 1.90**	**0.024**	**1.39**	**1.02; 1.90**	**0.039**	**2.21**	**1.28; 3.84**	**0.005**	**2.43**	**1.36; 4.34**	**0.003**
PfAMA1	1.49	0.99; 2.23	0.054	**1.65**	**1.06; 2.56**	**0.027**	**2.14**	**1.12; 4.09**	**0.021**	**2.10**	**1.11; 3.98**	**0.023**
PfEBA_175_	1.36	0.91; 2.02	0.134	1.31	0.86; 1.99	0.208	1.00	0.57; 1.76	0.987	1.02	0.59; 1.79	0.932
PfDBL3x	1.24	0.80; 1.92	0.338	1.29	0.80; 2.10	0.293	1.09	0.65; 1.83	0.736	1.09	0.64; 1.85	0.754
PfDBL5ε	1.27	0.88; 1.84	0.208	1.24	0.83; 1.87	0.295	1.36	0.88; 2.10	0.162	1.38	0.89; 2.12	0.148
PfDBL6ε	1.18	0.74; 1.86	0.487	1.34	0.81; 2.23	0.255	0.95	0.55; 1.64	0.857	0.94	0.54; 1.62	0.815

*Crude (adjusted only by site) and adjusted logistic regression models were estimated to calculate the odds ratio (OR) of having *Plasmodium vivax* (*Pv*) or *Plasmodium falciparum* (*Pf*) infection at delivery per doubling antibody levels (MFI) at recruitment. Variables in the adjusted analyses are country of origin, age, gravidity (number of previous gestations), and gestational age. Only uninfected women at recruitment were included in the analysis. *N*: crude analysis = 456 [*Pv* ligand Duffy binding protein (PvDBP)-inhibition = 121]; adjusted analysis = 445 (PvDBP-inhibition = 118). Highlighted in bold if *p*-value < 0.05*.

Subsequently, we assessed the association between antibody levels at recruitment and delivery outcomes (Hb and BW). Anti-PvCSP-C and anti-PvCSP IgG levels had a positive and negative association, respectively, with Hb levels at delivery, but these associations were not maintained in the adjusted analysis. Anti-PvCSP-C and anti-PfDBL3x antibody levels, and the percentage of inhibitory anti-PvDBP antibodies, were positively associated with BW (Table 5), but only the last two variables maintained the association in the adjusted analysis.

**Table 5:** **Association of antibody levels at recruitment with delivery outcomes**.

	Hemoglobin (g/dL)						BW (g)					
	Crude			Adjusted			Crude			Adjusted		
	Diff	95% CI	*p*	Diff	95% CI	*p*	Diff	95% CI	*p*	Diff	95% CI	*p*
PvCSP-N	0.0	−0.1; 0.2	0.640	0.0	−0.1; 0.2	0.639	6	−34; 46	0.771	10	−31; 51	0.634
PvCSP-C	**0.2**	**0.0; 0.4**	**0.045**	0.1	−0.1; 0.3	0.292	**47**	**0.6; 93**	**0.048**	37	−10; 84	0.121
PvCSP-R	0.1	−0.1; 0.2	0.361	0.0	−0.2; 0.2	0.966	28	−16; 72	0.209	27	−17; 71	0.230
PvCSP	**−0.1**	**−0.1; −0.0**	**0.046**	−0.1	−0.1; 0.0	0.118	−2	−19; 14	0.796	1	−16; 17	0.937
PvDBP	0.0	−0.1; 0.1	0.521	0.1	−0.1; 0.2	0.368	4	−22; 31	0.740	−9	−37; 20	0.544
PvMSP1_19_	0.0	−0.1; 0.0	0.322	0.0	−0.1; 0.0	0.531	13	−7; 33	0.201	9	−12; 30	0.406
Pv200L	0.0	−0.1; 0.1	0.544	0.0	−0.1; 0.1	0.888	−3	−27; 22	0.825	2	−23; 26	0.896
PvMSP1-N	0.0	−0.1; 0.0	0.627	0.0	−0.0; 0.0	0.800	7	−5; 18	0.267	−3	−16; 9	0.608
PvMSP5	0.0	−0.0; 0.0	0.951	0.0	−0.0; 0.0	0.973	2	−11; 14	0.795	−10	−23; 3	0.119
PvDBP-Inh	0.0	−0.1; 0.1	0.959	0.0	−0.1; 0.2	0.626	**49**	**8; 90**	**0.020**	**45**	**1; 89**	**0.046**
PfMSP1_19_	0.0	−0.1; 0.1	0.845	0.1	−0.0; 0.1	0.296	8	−14; 30	0.470	12	−10; 35	0.285
PfAMA1	0.0	−0.1; 0.1	0.876	0.1	−0.1; 0.1	0.378	10	−16; 36	0.447	18	−10; 45	0.205
PfEBA_175_	0.0	−0.1; 0.1	0.816	0.0	−0.1; 0.1	0.947	2	−31; 35	0.898	3	−32; 37	0.883
PfDBL3x	0.1	−0.0; 0.2	0.169	0.1	−0.0; 0.3	0.101	**63**	**26; 101**	**0.001**	**60**	**22; 99**	**0.003**
PfDBL5ε	0.0	−0.1; 0.1	0.750	0.1	−0.1; 0.2	0.320	24	−9; 56	0.157	17	−16; 49	0.323
PfDBL6ε	0.0	−0.1; 0.2	0.626	0.1	−0.0; 0.2	0.168	21	−14; 57	0.243	18	−18; 54	0.325

*Diff: difference in hemoglobin (Hb) at delivery (g/dL) or birth weight (BW, expressed in grams) per doubling antibody levels at recruitment. Highlighted in bold if *p* < 0.05. Regression analyses were performed adjusting by site, gestational age at recruitment (weeks), age (years), parity, Hb levels at recruitment (for Hb analysis at delivery), and *Plasmodium vivax* and *Plasmodium falciparum* infection. *N* = 382 [*Pv* ligand Duffy binding protein (PvDBP)-inhibition = 106]*.

### Antigen-Specific Cellular Immune Responses to *Pv* Antigens

**Figure 5:**
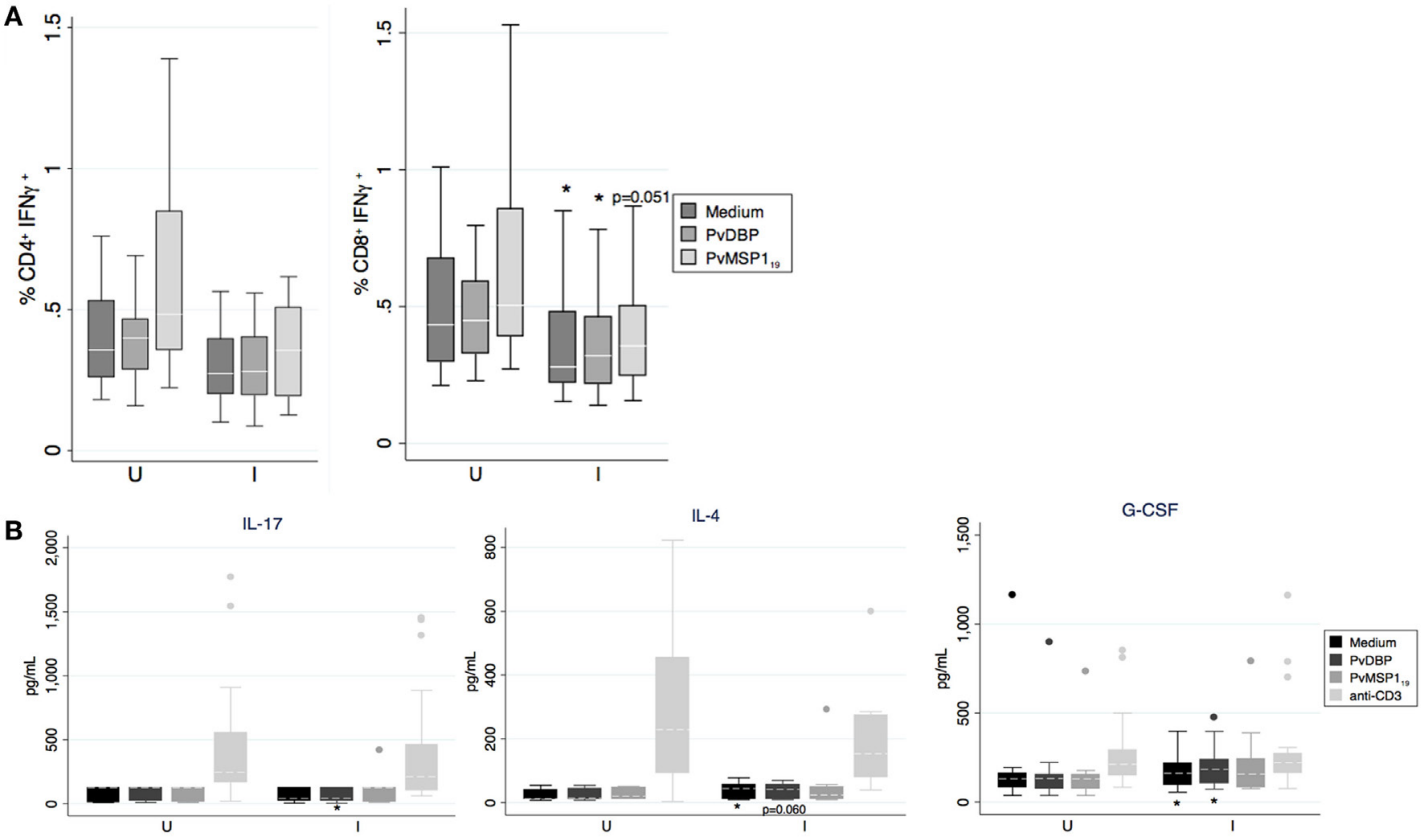
***In vitro* production of cytokines by peripheral blood mononuclear cell (PBMC) in response to antigen stimulation**. **(A)** Percentage of IFN-γ^+^ cells on CD4^+^ and CD8^+^ T cells as indicated by boxplot with whiskers or **(B)** cytokine concentration in supernatant after incubation of PBMCs with medium, Pv ligand Duffy binding protein (PvDBP) peptides, PvMSP1_19_, or anti-CD3 mAb were analyzed and compared between *Plasmodium vivax* infected (I, *N* = 28 except PvMSP1_19_ where *N* = 15) or uninfected women (U, *N* = 18 except PvMSP1_19_ where *N* = 12). Median (white line), and 25^th^ and 75^th^ percentiles (lower and upper hinge, respectively) are represented in the boxes. Outside values were excluded in panel **(A)**. *p < 0.05, Mann–Whitney test.

We compared the cellular immune responses to PvDBP peptides and PvMSP1_19_ between *Pv* infected and uninfected pregnant women in the PNG cohort. PBMCs from women with a concurrent *Pv* infection had less CD8^+^IFN-γ^+^ cells than uninfected women independently of whether we cultured the cells in the presence of the antigens or medium alone (Figure 5). The differences for the CD4^+^IFN-γ^+^ population were not statistically significant (medium *p* = 0.085, DBP *p* = 0.083, PvMSP1_19_ *p* = 0.064, Figure 5). No differences between infected and uninfected groups were found when we stimulated with anti-CD3 (24) or when we measured CD69^+^ T cells (not shown). In the supernatants of the PBMCs cultured with either PvDBP peptides (Table 6; Figure 5) or medium alone (24), we found significantly more G-CSF and IL-4 in the infected than in the uninfected group. Specifically in response to PvDBP peptides, PBMCs from infected women secreted significantly less IL-17 than those from uninfected women (Table 6; Figure 5). However, none of these differences remained significant after adjusting for multiple comparisons, and these results should be interpreted with caution.

**Table 6:** **Cytokine, chemokine, and growth factor concentrations in peripheral blood mononuclear cell supernatants**.

	DBP				PvMSP1_19_			
	U	I	*p*	adj-*p*	U	I	*P*	adj-*p*
EGF	40	40	0.983	1	32	40	0.263	1
CCL11	13	3	0.221	1	3	3	0.555	1
FGF-basic	18	17	0.812	1	15	17	0.945	1
G-CSF	**131**	**183**	**0.033**	0.99	129	157	0.212	1
GM-CSF	38	38	0.733	1	38	38	0.151	1
HGF	245	162	0.123	1	265	193	0.381	1
IFN-α	238	208	0.523	1	208	226	0.407	1
IFN-γ	13	13	0.772	1	13	13	0.481	1
IL-10	56	99	0.283	1	33	44	0.332	1
IL-12	71	62	0.610	1	52	38	0.963	1
IL-13	74	73	0.696	1	67	74	0.549	1
IL-15	147	152	0.801	1	282	127	0.709	1
IL-17	**125**	**42**	**0.046**	1	125	125	0.960	1
IL-1β	77	72	0.812	1	50	63	0.311	1
IL-1Ra	1,731	1,503	0.340	1	1,521	1,478	0.945	1
IL-2	11	9	0.137	1	12	8	0.213	1
IL-2R	209	235	0.665	1	100	134	0.140	1
IL-4	14	42	0.060	1	20	24	0.870	1
IL-5	8	8	0.234	1	8	8	0.739	1
IL-6	1,513	2,701	0.129	1	907	1,623	0.102	1
IL-7	75	76	0.182	1	75	75	0.979	1
CXCL-8	64,000	64,000	0.720	1	64,000	64,000	0.283	1
IP10	7	6	0.495	1	6	6	0.596	1
CCL2	18,010	16,677	0.811	1	15,351	19,126	0.627	1
CXCL9	113	113	0.320	1	113	113	0.832	1
CCL3	2,289	2,308	0.845	1	516	1,081	0.269	1
CCL4	1,840	1,881	0.633	1	978	1,168	0.214	1
CCL5	139	162	0.948	1	70	52	0.417	1
TNF	89	77	0.845	1	34	53	0.056	1
VEGF	90	111	0.335	1	63	94	0.134	1

*Median concentration (pg/mL) values for each cytokine, chemokine, and growth factor in supernatants from the infected (I, *N* = 28) and uninfected (U, *N* = 18) groups after stimulation with DBP peptides or PvMSP1_19_. Reference values for medium and anti-CD3 stimulation have been published previously (24). *p* corresponds to Mann–Whitney test. Highlighted in bold if *p* < 0.05. adj-*p* corresponds to the *p*-value adjusted for multiple comparisons using the Bonferroni method*.

*G-CSF, granulocyte colony-stimulating factor; GM-CSF, granulocyte macrophage colony-stimulating factor; IFN, interferon; IL, interleukin; TNF, tumor necrosis factor; IP, IFN-γ-inducible protein; EGF, epidermal growth factor; FGF, fibroblast growth factor; HGF, hepatocyte growth factor; VEGF, vascular endothelial growth factor*.

## Discussion

Here, we present data on immune responses to a large panel of *Plasmodium* antigens in pregnant women from five countries with different malaria transmission patterns followed up since recruitment at first antenatal clinic visit until postpartum. Responses to each antigen were very variable but, in general, antibody levels were quite consistent with recent patterns of malaria transmission intensity in the areas. Furthermore, we show evidence of impact of submicroscopic infections and cross-species reactivity on antibody responses, and positive effects of functional antibodies on delivery outcomes.

Women from PNG had the highest antibody responses for all antigens at recruitment and delivery, and this difference was more dramatic for anti-*Pf* responses, in agreement with the highest *Pf* prevalence. PNG is also the country with the highest endemicity for *Pv*, even if at the time of the study there was a transient drop in malaria transmission intensity there. Nevertheless, at postpartum, women from CO had higher IgG levels than PNG for most anti-*Pv* antigens, which can be explained by a very high prevalence of *Pv* infections (40%) in our cohort in CO at postpartum (24), which was not observed to that extent at recruitment or delivery. BR and IN presented intermediate and low anti*-Pv* IgG levels, respectively, and very low anti-*Pf* responses, in agreement with their infection prevalence rates.

We observed different sera reactivity for full-length PvCSP and its peptides between PNG and the other countries. This may be explained by different distribution of the two genetic variants of the PvCSP repeat region in the different malaria regions worldwide. The full-length protein was a chimeric one with the two variants (VK247 and VK210), whereas the R peptide tested was based on the VK210. In previous studies in sera from Colombia subjects, it was showed a higher recognition of VK210 (46) than of VK247, which would explain the high reactivity of the peptide R at postpartum in CO. Of note, although anti-PvDBP levels were not high in BR, they showed the highest percentage of binding inhibition to Duffy antigen. Differences in the level of inhibition have been associated to the Duffy antigen phenotype (47, 48) but whether women from BR express the protective Duffy phenotype in a higher proportion than women from the other countries could not be assessed in this study. Regardless, our results support the previous finding that efficacy of a PvDBP-based vaccine may differ among populations (47).

Antibody patterns in GT were very interesting. There were remarkable anti-*Pv* humoral responses despite very low *Pv*-infection prevalence by microscopy but in agreement with the high prevalence of submicroscopic infections in the country, as shown in this cohort (24) and in a different study (49). Plasma antibodies are a combined result of those secreted by long-lived plasma B cells (from previous exposures) and those boosted by memory B cells in each new infection. On the one hand, although antibody titers are generally perceived to decline rapidly in the absence of reinfection, half-lives of 4 years for PvAMA1 have been reported in pregnant women (12). On the other hand, our results also suggest that submicroscopic infections are enough to boost *Plasmodium*-specific antibody responses, emphasizing the importance of analyzing submicroscopic infections in cohort studies, especially in adult populations and/or low-transmission areas.

Surprisingly, we found more than 60% of responders for PfMSP1_19_ and PfAMA1 in GT, despite low *Pf*-infection prevalence both by microscopy and PCR. Historic exposure surely accounts for this finding, as half-lives of 4–10 years for PfMSP1_19_ and PfAMA1 have been reported (12, 50). Nevertheless, we also found that coinfected individuals had more anti-*Pf* antibodies than individuals with *Pf*-monoinfection, thus it would be possible that *Pv* infections boosted anti-*Pf* responses in GT. Both MSP1 and AMA1 have orthologs in *Pv* and *Pf*, and we found a high level of correlation between *Pv* and *Pf* anti-merozoite levels. However, although species cross-reactivity has been described for other antigens such as MSP5 (51) and CLAG (52), it was not observed with MSP1 (53), and it has not been described for AMA1. An alternative explanation could be a *Pf*-specific B cell bystander activation by the pro-inflammatory *Pv* infection (54).

IgGs against PvCSP peptides and PfDBL3x were higher at postpartum compared to recruitment and delivery, but no changes at postpartum were observed for the remaining antibodies. In accordance, previous manuscripts have shown increases (55–57), no changes (58) and even decreases (59) in *Pf* and *Pv* antibody levels at postpartum compared to pregnancy, depending on study site and antigen. We also believe the timing of sampling after delivery could help explain some of the differences observed in the literature. Therefore, there is a need to harmonize immune-epidemiological studies at postpartum to elucidate whether this is a period of immunological recovery or susceptibility.

Although many of the antigens studied here are vaccine candidates, no protective association with future *Plasmodium* infection was found. On the contrary, a positive (non-protective) association was found with some antibody levels at recruitment and future infection, as previously described in a cohort in Thailand (12), suggesting that these antibodies are markers of exposure in our cohort. Actually, this was not entirely surprising as women are known to be susceptible to *Pf* infections during pregnancy despite having acquired immunity (19). In addition, studies showing protection for *Pf* antigens such as PfMSP1_19_ or PfAMA1 have been conducted mainly in children/teenagers (60), and we and others have previously reported lack of protective associations in adults (51, 61–64). Moreover, it has already been reported in a number of studies that one of the strongest risk factors for falciparum malaria is having had episodes in the past (65, 66). However, it is not clear whether anti-*Pv* antibodies confer protection for *Pv* malaria during pregnancy. Even though our data suggest that they do not, a limitation in this study, which is an unsolved and highly complex issue in most field studies, was how to control the heterogeneity of previous exposure at the individual level and thus distinguish when antibodies are markers of exposure or protection.

In this line, we observed no association between anti-VAR2CSA responses and gravidity or protection against future *Pf* infection, in contrast to many studies in Africa but similarly to findings in Thailand (12). In fact, it has been postulated that multigravidae are likely to be serologically equivalent to primigravidae in areas of low transmission (67). It is also possible that we could not find a protective association with infection because (a) active detection was only performed at recruitment and delivery, so we might have missed asymptomatic infections during pregnancy; (b) most of the infections were submicroscopic for which naturally acquired immunity does not confer sterile protection. Importantly, submicroscopic *Pf* infections are still associated with poor outcomes like anemia and low BW even if they are asymptomatic [(68) and unpublished results]. In this sense, we found a positive protective association between anti-PfDBL3x IgG levels at recruitment and BW, as previously shown in a few studies (23, 67).

Interestingly, we report for the first time to our knowledge a positive association between the levels of binding-inhibitory anti-PvDBP antibodies at recruitment and BW. This result was somehow unexpected as we did not observe protection against future *Pv* infection, neither did we observe an association between *Pv* infection and low BW (LBW, <2,500 g) in the main Pregvax cohort (unpublished results). High functional (inhibitory) anti-PvDBP levels have been associated with delayed time to *Pv* reinfection, reduced risk of *Pv*, and reduction in *Pv* parasitemia in children from PNG (29). Furthermore, levels of functional but not general anti-PvDBP antibodies have been associated with protection against clinical malaria in adults in Brazil (30). It seems that protection against infection is only observed in children or for symptomatic malaria, which could explain why we did not observe an association with infection in our cohort. Regardless, our results suggest that functional anti-PvDBP might protect against deleterious effects of *Pv* malaria during pregnancy. Further studies are needed to confirm this effect.

Regarding cellular immune responses, we observed differences between infected and non-infected women, but these did not seem to be antigen-specific as they were observed also when PBMCs were cultured with medium alone. First, we believe that baseline immune status and non-specific immune responses may have a significant effect on adaptive immune responses and morbidity outcomes. Second, as most of the infections in our cohort were only detected by PCR, this may imply that high parasitemia infections are necessary to boost antigen-specific cellular responses for these two antigens, contrary to the antibody responses. In our study, it seems that the pregnancy status favoring a systemic switch to a T_H_2 response (69) or type-2 immunity in general (70) may limit any T_H_1 parasite-specific protective response. In that sense, in the infected group, independently of the stimulus, we observed a lower percentage of intracellular IFN-γ (only significant for CD8^+^ T cells) and higher secretion of IL-4 (a key T_H_2 cytokine) and G-CSF, which has been shown to induce T_H_2 polarization in CD4^+^ T cells (71). Although we found differences in the percentage of IFN-γ^+^ T cells between infected and uninfected women, this did not translate into differences in the levels of secreted IFN-γ. It is likely that the short incubation period of the PBMCs after antigen stimulation (12 h until secretion was stopped) might have been insufficient for eliciting a significant detectable cumulative change of the cytokine in culture supernatants. Moreover, other IFN-γ cellular sources like NK cells may counteract the T cell effect.

In summary, we have investigated the immune responses to several malaria vaccine candidates in a large and geographically diverse cohort of pregnant women. Our data suggest that submicroscopic infections are enough to boost naturally acquired antibody responses to malaria but maybe not T cell responses. We also show that antibody responses to several malaria vaccine candidates appear to be boosted in coinfections or in women infected with the other *Plasmodium* species studied, which might be interesting in programmatic terms regarding the search for a malaria vaccine. Importantly, we report that inhibitory anti-PvDBP antibody levels have a positive association with BW, suggesting that acquisition of functional anti-PvDBP antibodies might confer protection against one poor outcome of malaria in pregnancy.

## Author Contributions

Substantial contributions to the conception or design of the work: CM, CD, HP, AB, AMay, IM, SR, and MD. Acquisition of samples/data: NP, FM-E, MC, CB-M, AM, MA-H, SK, SKK, DK, LR, and PS. Analysis of samples: PR, DH, CC, MM, and CS. Analysis of data: SS, LQ, PR, and DH. Interpretation of data for the work and drafting the manuscript: PR and CD. Revising it critically for important intellectual content; final approval of the version to be published; and agreement to be accountable for all aspects of the work in ensuring that questions related to the accuracy or integrity of any part of the work are appropriately investigated and resolved: PR, MA-H, MM, FM-E, NP, CB-M, AM, DH, MC, LR, PS, SK, SKK, DK, MD, SS, LQ, AMay, SR, IM, CS, HP, AB, CC, CM, and CD.

## Disclaimer

The findings and conclusions presented in this manuscript are those of the authors and do not necessarily reflect the official position of the U.S. Centers for Disease Control and Prevention.

## Conflict of Interest Statement

The authors declare that the research was conducted in the absence of any commercial or financial relationships that could be construed as a potential conflict of interest.
