# Supplementary material for: Naturally Acquired Binding-Inhibitory Antibodies to Plasmodium vivax Duffy Binding Protein in Pregnant Women Are Associated with Higher Birth Weight in a Multicenter Study
Source: Front Immunol. 2017 Feb 17;8:163. doi: 10.3389/fimmu.2017.00163 (PMC5313505; doi:10.3389/fimmu.2017.00163)
Supplement: Supplementary file 1 [file table_1.docx]

Supplementary Material

Naturally Acquired Binding-Inhibitory Antibodies to *Plasmodium vivax* Duffy Binding Protein in Pregnant Women Are Associated with Higher Birth Weight in a Multicenter Study

Pilar Requena^1,*,^ Myriam Arévalo-Herrera^2^, Michela Menegon^3^ , Flor E. Martínez-Espinosa^4,5^, Norma Padilla^6^, Camila Bôtto-Menezes^5,7^, Adriana Malheiro^8^, Dhiraj Hans^9^, Maria Eugenia Castellanos^6^, Leanne Robinson^10,11,12^, Paula Samol^10^, Swati Kochar^13^, Sanjay K. Kochar^13^, Dhanpat K. Kochar^13^, Meghna Desai^14^, Sergi Sanz^1^, Llorenç Quintó^1^, Alfredo Mayor^1^, Stephen Rogerson^15^, Ivo Mueller^1,12^, Carlo Severini^3^, Hernando del Portillo^1,16^, Azucena Bardají^1^, Chetan C. Chitnis^9^, Clara Menéndez^1^, Carlota Dobaño^1,*^

*** Correspondence:** Corresponding Author: [pilar.requena.mendez@gmail.com](mailto:pilar.requena.mendez@gmail.com), carlota.dobano@isglobal.org

# Supplementary Table

Mixed-effect multi level regressions were estimated (PD: proportional difference). Timepoint was estimated as the fixed independent variable, and inter-site (country of origin) and inter-subject variability were estimated as random parts (not shown).

|  | **Fixed part** | | | | |
| --- | --- | --- | --- | --- | --- |
|  | **Timepoint** | **PD** | **95% CI** | **p-value** | **p Wald** |
| **PvCSP-N** | **Recruitment** | **1** | **-** | **-** | **0.0276** |
|  | **Delivery** | **1.02** | **0.90; 1.17** | **0.7205** |  |
|  | **Postpartum** | **1.21** | **1.05; 1.40** | **0.0104** |  |
| **PvCSP-C** | **Recruitment** | **1** | **-** | **-** | **0.0337** |
|  | **Delivery** | **0.95** | **0.83; 1.08** | **0.4512** |  |
|  | **Postpartum** | **1.15** | **1.00; 1.33** | **0.0517** |  |
| **PvCSP-R** | **Recruitment** | **1** | **-** | **-** | **< 0.0001** |
|  | **Delivery** | **0.88** | **0.77; 1.01** | **0.0678** |  |
|  | **Postpartum** | **1.32** | **1.14; 1.53** | **0.0003** |  |
| PvCSP | Recruitment | 1 | - | - | 0.7743 |
|  | Delivery | 1.12 | 0.79; 1.59 | 0.5104 |  |
|  | Postpartum | 1 | 0.68; 1.47 | 0.9835 |  |
| PvDBP | Recruitment | 1 | - | - | 0.158 |
|  | Delivery | 1.07 | 0.97; 1.18 | 0.1956 |  |
|  | Postpartum | 1.15 | 0.98; 1.34 | 0.0812 |  |
| PvMSP1_19_ | Recruitment | 1 | - | - | 0.2296 |
|  | Delivery | 1.04 | 0.93; 1.17 | 0.4949 |  |
|  | Postpartum | 1.17 | 0.98; 1.40 | 0.0878 |  |
| Pv200L | Recruitment | 1 | - | - | 0.1196 |
|  | Delivery | 0.94 | 0.86; 1.03 | 0.1787 |  |
|  | Postpartum | 1.08 | 0.94; 1.24 | 0.2817 |  |
| PvMSP1-N | Recruitment | 1 | - | - | 0.6969 |
|  | Delivery | 1.22 | 0.75; 2.01 | 0.4249 |  |
|  | Postpartum | 1.18 | 0.69; 2.04 | 0.5463 |  |
| PvMSP5 | Recruitment | 1 | - | - | 0.7898 |
|  | Delivery | 1.01 | 0.72; 1.43 | 0.9522 |  |
|  | Postpartum | 1.14 | 0.77; 1.67 | 0.5179 |  |
| **PfMSP1_19_** | **Recruitment** | **1** | **-** | **-** | **0.0064** |
|  | **Delivery** | **0.89** | **0.83; 0.96** | **0.0035** |  |
|  | **Postpartum** | **1.02** | **0.90; 1.15** | **0.7714** |  |
| PfAMA-1 | Recruitment | 1 | - | - | 0.7851 |
|  | Delivery | 0.98 | 0.91; 1.06 | 0.6431 |  |
|  | Postpartum | 1.02 | 0.90; 1.16 | 0.725 |  |
| PfEBA_175_ | Recruitment | 1 | - | - | 0.1837 |
|  | Delivery | 1 | 0.94; 1.07 | 0.9737 |  |
|  | Postpartum | 1.09 | 0.99; 1.21 | 0.0816 |  |
| **PfDBL3x** | **Recruitment** | **1** | **-** | **-** | **0.0001** |
|  | **Delivery** | **0.98** | **0.91; 1.05** | **0.5531** |  |
|  | **Postpartum** | **1.23** | **1.11; 1.37** | **0.0001** |  |
| PfDBL5ε | Recruitment | 1 | - | - | 0.6091 |
|  | Delivery | 1.03 | 0.90; 1.18 | 0.6419 |  |
|  | Postpartum | 1.08 | 0.93; 1.25 | 0.3203 |  |
| PfDBL6ε | Recruitment | 1 | - | - | 0.7014 |
|  | Delivery | 1.03 | 0.90; 1.18 | 0.6663 |  |
|  | Postpartum | 1.07 | 0.92; 1.24 | 0.4025 |  |
